# Simulated Microgravity Induces Regionally Distinct Neurovascular and Structural Remodeling of Skeletal Muscle and Cutaneous Arteries in the Rat

**DOI:** 10.3389/fphys.2020.00675

**Published:** 2020-06-30

**Authors:** Olga S. Tarasova, Vjatcheslav U. Kalenchuk, Anatoly S. Borovik, Veronika O. Golubinskaya, Michael D. Delp, Olga L. Vinogradova

**Affiliations:** ^1^ State Research Center of the Russian Federation, Institute of Biomedical Problems, Russian Academy of Sciences, Moscow, Russia; ^2^ Faculty of Biology, M.V. Lomonosov Moscow State University, Moscow, Russia; ^3^ Faculty of Basic Medicine, M.V. Lomonosov Moscow State University, Moscow, Russia; ^4^ Department of Nutrition, Food and Exercise Sciences, College of Human Sciences, Florida State University, Tallahassee, FL, United States

**Keywords:** microgravity, hindlimb unloading, rat, small arteries, remodeling, sympathetic innervation, noradrenaline, serotonin

## Abstract

**Introduction**: Mechanical forces and sympathetic influences are key determinants of vascular structure and function. This study tested the hypothesis that hindlimb unloading (HU) exerts diverse effects on forelimb and hindlimb small arteries of rats in functionally different regions of the skeletal muscle and skin.

**Methods**: Male Wistar rats were subjected to HU for 2 weeks, then skeletal muscle arteries (deep brachial and sural) and skin arteries (median and saphenous) were examined *in vitro* using wire myography or isobaric perfusion and glyoxylic acid staining.

**Results**: HU increased lumen diameter of both forelimb arteries but decreased diameter of the sural artery; the saphenous artery diameter was not affected. Following HU, maximal contractile responses to noradrenaline and serotonin increased in the forelimb but decreased in the hindlimb skeletal muscle feed arteries with no change in skin arteries; all region-specific alterations persisted after endothelium removal. HU increased the sensitivity to vasoconstrictors in the saphenous artery but not in the sural artery. In the saphenous artery, initially high sympathetic innervation density was reduced by HU, sparse innervation in the sural artery was not affected. Electrical stimulation of periarterial sympathetic nerves in isobarically perfused segments of the saphenous artery demonstrated a two-fold decrease of the contractile responses in HU rats compared to that of controls.

**Conclusion**: HU induces contrasting structural and functional adaptations in forelimb and hindlimb skeletal muscle arteries. Additionally, HU had diverse effects in two hindlimb vascular regions. Hyper-sensitivity of the saphenous artery to vasoconstrictors appears to result from the shortage of trophic sympathetic influence. Importantly, HU impaired sympathetically induced arterial vasoconstriction, consistent with the decreased sympathetic constrictor response in humans following space flight.

## Introduction

The vascular system is constantly exposed to mechanical forces such as transmural pressure and shear stress, the latter of which is created by flowing blood on the endothelial cell lining of the vessel. Prolonged changes in these factors govern gross structural remodeling of blood vessels, which in turn affects their functional characteristics. For example, previous work has shown that chronic increases in transmural pressure induce hypertrophy of the vascular wall and increases in vessel contractility, while decreases in pressure result in the opposite changes (Folkow, 1982). Chronic increases in shear stress, in turn, lead to structural increases in maximal diameter, whereas decreases in shear result in a narrowing of the vessel lumen (Langille, 1993; Pourageaud and De Mey, 1997).

The cardiovascular system of all terrestrial inhabitants is adapted to the gravitational field of the Earth and changes significantly in conditions of weightlessness (Watenpaugh and Hargens, 1996). Importantly, redistribution of transmural pressures and flows within the arterial vasculature during space flight or simulated microgravity is known to initiate different adaptations in vessels from different anatomic regions (Zhang, 2001, 2013). In humans, exposure to long term head-down bed rest was shown to reduce intimal-medial thickness and lumen diameter of arterial vessels in lower extremities, in contrast to upper extremity and cranial arteries (Bleeker et al., 2005; Platts et al., 2009).

The model of hindlimb unloading (HU) in rats is commonly used to study the mechanisms of microgravity effects on different body systems, including vasculature (Globus and Morey-Holton, 2016). Even relatively small change in hydrostatic pressure gradient, as observed in the rat body with this model (Colleran et al., 2000), results in different vascular alterations in rostral and caudal parts of the body. Similar to microgravity effects in human vascular system, rat hindlimb arteries commonly demonstrate wall thinning and lumen reduction, in contrast to cranial arteries (Wilkerson et al., 1999; Delp et al., 2000; Sun et al., 2004; Stabley et al., 2013).

Data from the literature also provide the evidence that transmural pressure shifts cannot explain all effects of simulated microgravity on vascular characteristics, because vascular alterations in HU rats may differ in functionally different vascular beds within the same part of the body. This was shown in studies of vascular dimensions and reactivity of arterioles from postural (soleus) and phasic (gastrocnemius) hindlimb muscles (Delp et al., 2000), as well as arterioles from white (glycolytic) and red (oxidative) parts from the same gastrocnemius muscle (Heaps and Bowles, 2002) of HU rats. Obviously, such diverse effects of HU on the arterial vasculature within and among postural and phasic muscles are largely the result of differential alterations in muscle activity and blood flow during the conditions of disuse (McDonald et al., 1992).

Structural and functional changes in the vascular bed could also result from the influence of sympathetic nervous system. In addition to acute control of vessel diameter and blood flow, neurotransmitters from the sympathetic nerves exert a trophic effect on the vasculature (Bevan, 1984; Puzdrova et al., 2014; Adeoye et al., 2015). The severity of trophic sympathetic influence in certain vascular bed may depend on the density of its innervation and/or on the efferent sympathetic traffic it receives. In addition, the state of the vascular neuroeffector apparatus has been shown to depend on the prevailing level of transmural pressure and, therefore, may be affected in the conditions of microgravity (Monos et al., 2001; Zhang, 2001; Puzdrova et al., 2009).

In contrast to the skeletal muscle circulation, the cutaneous circulation is regulated by a smaller contribution from metabolic vasomotor mechanisms and a higher role of sympathetic control, including a greater sympathetic vasoconstriction of small cutaneous arteries relative to that in skeletal muscle (Tarasova et al., 2003). However, to our knowledge, no study has examined the effects of simulated microgravity *via* HU on the structural and functional properties of cutaneous arteries, nor contrasted these effects in the forelimb and hindlimb circulations. Therefore, this study tested the hypothesis that HU exerts diverse effects on the neurovascular density, structural properties, and functional vasoconstrictor responsiveness of skeletal muscle and cutaneous feed arteries in the rat forelimb and hindlimb regions.

## Materials and Methods

All the procedures were conducted in accordance with the European Convention for the Protection of Vertebrate Animals used for experimental and other scientific purposes and conformed to the Guide for the Care and Use of Laboratory Animals published by the US National Institutes of Health (Eighth edition, 2011). The procedures were approved by the Biomedical Ethics Committee of the Institute of Biomedical Problems, Russian Academy of Sciences (protocol N224).

### Animals

Male Wistar rats (2.5–3-month-old) were obtained from the breeder of the Institute of Biomedical Problems and housed in laboratory animal unit at the Faculty of Biology, M.V. Lomonosov Moscow State University in controlled conditions of temperature (22–24°C) and light-dark cycle (12–12 h) with *ad libitum* access to water and standard chow diet (Laboratorkorm, Russia). After 1-week acclimatization to the environment, the rats were randomly assigned to the cage control (*n* = 10) and HU (*n* = 10) groups. In HU group, the hindlimbs of the rat were elevated with a hook attached to the proximal third of the tail with adhesive material. The hook was connected by a harness to a swivel apparatus at the top of the cage. The height of the hindquarter elevation was adjusted to prevent the hindlimbs from touching the supporting surfaces, resulting in a suspension angle of 35–40°. The forelimbs maintained the contact with the floor surface, which allowed the animals full range of motion. The animals were housed individually in cages of 50 cm × 50 cm × 50 cm. Animals remained under HU or control conditions for a total of 14 days. This duration of HU was shown to provide the stable changes in muscle weight and blood flow in rats (McDonald et al., 1992). No signs of ischemia or damage of the tail skin were observed during HU.

After 2 weeks of HU, the animals were sacrificed by decapitation under CO_2_ anesthesia and the arteries were dissected free from surrounding tissue. Two arteries were isolated from the forelimb (deep brachial artery and median artery) and two arteries from the hindlimb (sural artery and saphenous artery). These feed arteries provide blood flow to skeletal muscle (brachial and sural arteries) and predominantly to the skin (median and saphenous arteries). Brachial and median arteries were dissected from the right forelimb for myography experiments. Sural arteries from right and left hindlimbs were used for myography experiments and histochemical examination, respectively. Right saphenous artery from each rat was used for wire myography (distal part) and histochemical examination (proximal part), while the artery from the left hindlimb was studied in perfusion experiments.

### Wire Myography

Two neighboring segments with a length of 2 mm were cut from each type of artery and mounted in wire myograph system (DMT, Denmark, 410A or 620 model) for isometric force recoding at well-defined internal circumferences. Endothelium was gently removed from one of the segments by rubbing with a rat whisker. The preparations were kept at 37°C in physiological salt solution containing (in mM): 120 NaCl, 26 NaHCO_3_, 4.5 KCl, 1.2 NaH_2_PO_4_, 1.0 MgSO_4_, 1.6 CaCl_2_, 5.5 D-glucose, 0.025 EDTA, and 5 HEPES, equilibrated with gas mixture 5% CO_2_ + 95% O_2_ to maintain pH 7.4. Force readings were continuously recorded at 10 Hz sampling rate using E14-140 analog-to-digital data converter (L-Card, Russia) and PowerGraph 3.3 software (DISoft, Russia). Fully relaxed arterial segments were gradually stretched to d_100_, the inner diameter equivalent to a transmural pressure of 100 mmHg, and then set to 0.9d_100_, where maximal active force is developed (Mulvany and Halpern, 1977).

The arteries were activated three times with noradrenaline (10^−6^ M, Sigma). Functional integrity of the endothelium was checked by application of acetylcholine (10^−6^ M, Sigma) on top of noradrenaline-induced contraction (3 × 10^−7^ M). Endothelium-intact preparations relaxed in response to acetylcholine by at least 50% of the precontraction level and the relaxation response of endothelium-denuded preparations was not larger than 10%. The experimental protocol included consecutively performed concentration-response relationships to noradrenaline (10^−8^ – 3 × 10^−5^ М, Sigma) and serotonin (3 × 10^−8^ – 3 × 10^−5^ М, Sigma) with 30-min washout interval between them. The agonists were given cumulatively in half-log increments. The responses to noradrenaline were studied in the presence of propranolol (10^−6^ M, Sigma), to prevent relaxation mediated by β-adrenoceptors.

During data analysis, active force values were calculated by subtracting the passive force value (recorded in the preparation with fully relaxed smooth muscle) from all recorded force values (before the first and at each noradrenaline or serotonin concentration). Then, respective values of active wall tension were calculated as T=F/2l, where *T* is tension, *F* is active force and *l* is the segment length. The concentration-response relationships were fitted to a sigmoidal function with variable slope using GraphPad Prism 7.0 software (La Jolla, CA, USA) to calculate pD_2_ values (the negative logarithm of EC_50_). Inner diameter of each artery (d_100_) was estimated from its passive length-tension relationship.

### Constant-Pressure Perfusion

A segment of the saphenous artery was isolated, placed into the tissue bath, and cannulated at both ends. The vessel fragment length between the cannulae was about 5–6 mm. Krebs-Henseleit physiological salt solution was used for perfusion and superfusion (NaCl 119 mM, KCl 4.7 mM, CaCl_2_ 2.5 mM, MgSO_4_ 1.17 mM, NaHCO_3_ 25 mM, KH_2_PO_4_ 1.18 mM, D-glucose 5.5 mM, EDTA 0.026 mM, 37°C, and 95% O_2_ + 5% CO_2_). Artery was perfused under a constant pressure; input and output pressures were measured continuously using two pressure transducers (BLPR2, World Precision Instruments, USA) and kept at 60 and 50 mmHg, respectively. Volume flow rate was measured with 1N type transducer connected to transit-time flowmeter (Transonic Systems Inc., USA). All parameters were permanently recorded at 20 Hz sampling rate using E14-140 analog-to-digital data converter (L-Card, Russia) and PowerGraph 3.3 software (DISoft, Russia).

Intramural nerves were stimulated with rectangular electric pulses of changing polarity, with amplitude of 200–300 mA and 0.2 ms duration. All the effects of electrical stimulation could be blocked by tetrodotoxin (3 μM, Sigma), thus indicating the neurogenic origin of the response. Electric pulse frequency was 4, 8, or 12 Hz, the stimulation lasted for 30 s, and 3-min intervals were applied between the stimulations. Data analysis was performed using a custom program working under MATLAB (MathWorks Inc., USA). The constrictor response was evaluated as a relative change of the intralumen diameter (*D*), which was calculated by Poiseuille equation: $D=\sqrt[{4}]{\left(l\cdot Q\right)/\Delta P}$, *l*, length of the vessel segment between canulae; *Q*, volume flow rate; and Δ*P*, the difference between input and output pressures.

### Glyoxylic Acid Staining for Periarterial Adrenergic Innervation

Segments of saphenous and sural arteries were cut lengthwise and placed in 0.1 M PBS (pH 7.2) supplemented with 2% glyoxylic acid, 10% sucrose, and 0.03% Pontamine Sky Blue (Puzdrova et al., 2009). After a 30-min incubation, the preparation was flattened on the slide with the adventitia upward, dried (30 min in jet of warm air and 5 min at 100°C), and overlaid with mineral oil and covered with a cover glass.

A LUMAM R3 microscope (LOMO, USSR; eyepiece ×7, objective lens ×40) was used for visualization. The exciting light wavelength was 380–440 nm; the luminescence wavelength was 480–700 nm. The plexus density was estimated with a grid that covered a field of 300 × 300 μm on the preparation and consisted of 24 rows, each containing 22–23 round markers (the ratio of marker diameter to space between markers was 1:2.5). Counting was performed in three randomly selected fields and the results were averaged.

### Statistical Data Analysis

Statistical analysis was performed in GraphPad Prism 7.0. The normality of the data distribution was confirmed using D’Agostino-Pearson test. Unpaired Student’s *t*-test or Repeated Measures ANOVA with Bonferroni *post hoc* test was used, as appropriate. Statistical significance was reached at *p* < 0.05. All data are given as mean ± S.E.M. and *n* represents the number of animals.

## Results

### Body Weight

At the beginning of the experiment, body weights of rats from control and HU groups were 316 ± 6 g (*n* = 10) and 310 ± 9 g (*n* = 10), respectively (*p* > 0.05). Within 2 weeks, control group increased body weight to 376 ± 14 g (*n* = 10) and HU group to 353 ± 6 g (*n* = 10). No difference in body weight was observed between the two experimental groups at the end of the experiment (*p* > 0.05, control vs. HU).

### Inner Diameter of the Arteries

Both forelimb arteries from HU rats had larger inner diameters compared to the respective arteries from control rats (Table 1). However, when calculated as percentage of the mean value in control group, the increment of diameter in the brachial artery was more prominent compared to the median artery: 22.5 ± 3.0 and 10.2 ± 3.8%, respectively (*p* < 0.05).

**Table 1:** Relaxed inner diameter (d_100_, μm) of endothelium-intact forelimb and hindlimb arteries from control and hindlimb-unloaded (HU) rats.

Organ	Arteries	Control	Hindlimb unloaded
Forelimb	Brachial (*n* = 9; 9)	213 ± 15	261 ± 6^*^
	Median (*n* = 10; 10)	375 ± 10	413 ± 14^*^
Hindlimb	Sural (*n* = 10; 10)	250 ± 11	208 ± 11^*^
	Saphenous (*n* = 10; 9)	418 ± 20	424 ± 20

The numbers in parentheses indicates the numbers of animals in the groups.

* *p* < 0.05 HU vs. control.

In contrast, sural arteries of HU rats demonstrated a decrease of inner diameter (by 17.9 ± 4.7%) compared to the arteries of control rats (Table 1). The diameter of saphenous artery was not affected by HU (Table 1).

**Table 2:** Sensitivity to noradrenaline and serotonin (pD_2_ values) of endothelium-intact (Endo+) and endothelium-denuded (Endo−) hindlimb and forelimb arteries from control and HU rats.

Arteries		Noradrenaline		Serotonin	
		Control	Hindlimb unloaded	Control	Hindlimb unloaded
Brachial	Endo (+)	5.93 ± 0.14	5.93 ± 0.13	6.02 ± 0.06	6.10 ± 0.05
	Endo (−)	5.71 ± 0.22	6.05 ± 0.13	5.92 ± 0.07	5.97 ± 0.06
Median	Endo (+)	n.d.	n.d.	6.34 ± 0.14	6.45 ± 0.07
	Endo (−)	n.d.	n.d.	5.99 ± 0.15	6.01 ± 0.09
Sural	Endo (+)	6.18 ± 0.05	5.90 ± 0.13	5.97 ± 0.10	5.86 ± 0.10
	Endo (−)	6.07 ± 0.07	5.95 ± 0.16	5.97 ± 0.13	5.77 ± 0.07
Saphenous	Endo (+)	5.50 ± 0.08	5.77 ± 0.10^*^	6.00 ± 0.09	6.44 ± 0.14^*^
	Endo (−)	5.50 ± 0.08	5.82 ± 0.09^*^	5.99 ± 0.08	6.45 ± 0.18^*^

**Figure 1:**
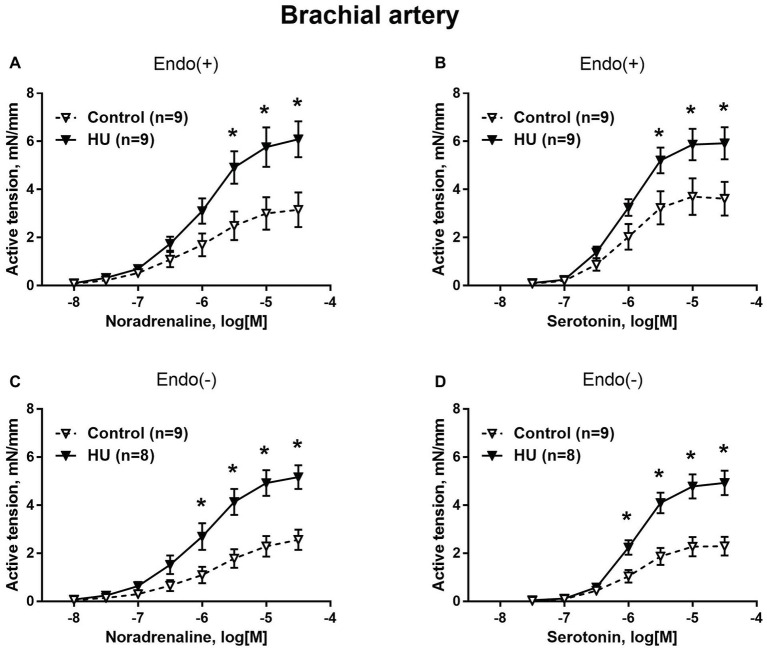
HU increases contractile responses of skeletal muscle feed arteries from the rat forelimb. Concentration-response relationships to noradrenaline **(A,C)** and serotonin **(B,D)** of endothelium-intact **(A,B)** and endothelium-denuded **(C,D)** deep brachial arteries in control and HU rats. The number in parentheses indicates the number of animals in the group. ^*^ *p* < 0.05 HU vs. control.

**Figure 2:**
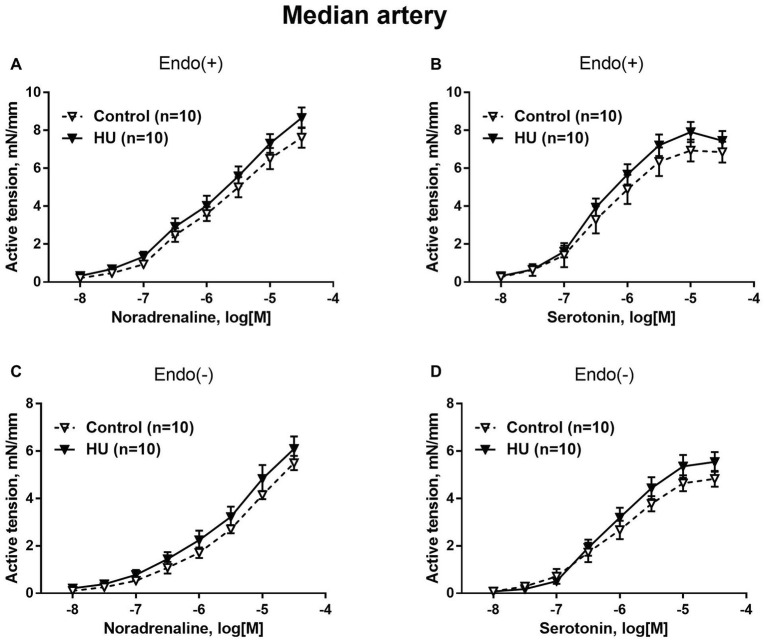
HU does not change contractile responses of skin feed arteries from the rat forelimb. Concentration-response relationships to noradrenaline **(A,C)** and serotonin **(B,D)** of endothelium-intact **(A,B)** and endothelium-denuded **(C,D)** median arteries in control and HU rats. The number in parentheses indicates the number of animals in the group.

**Figure 3:**
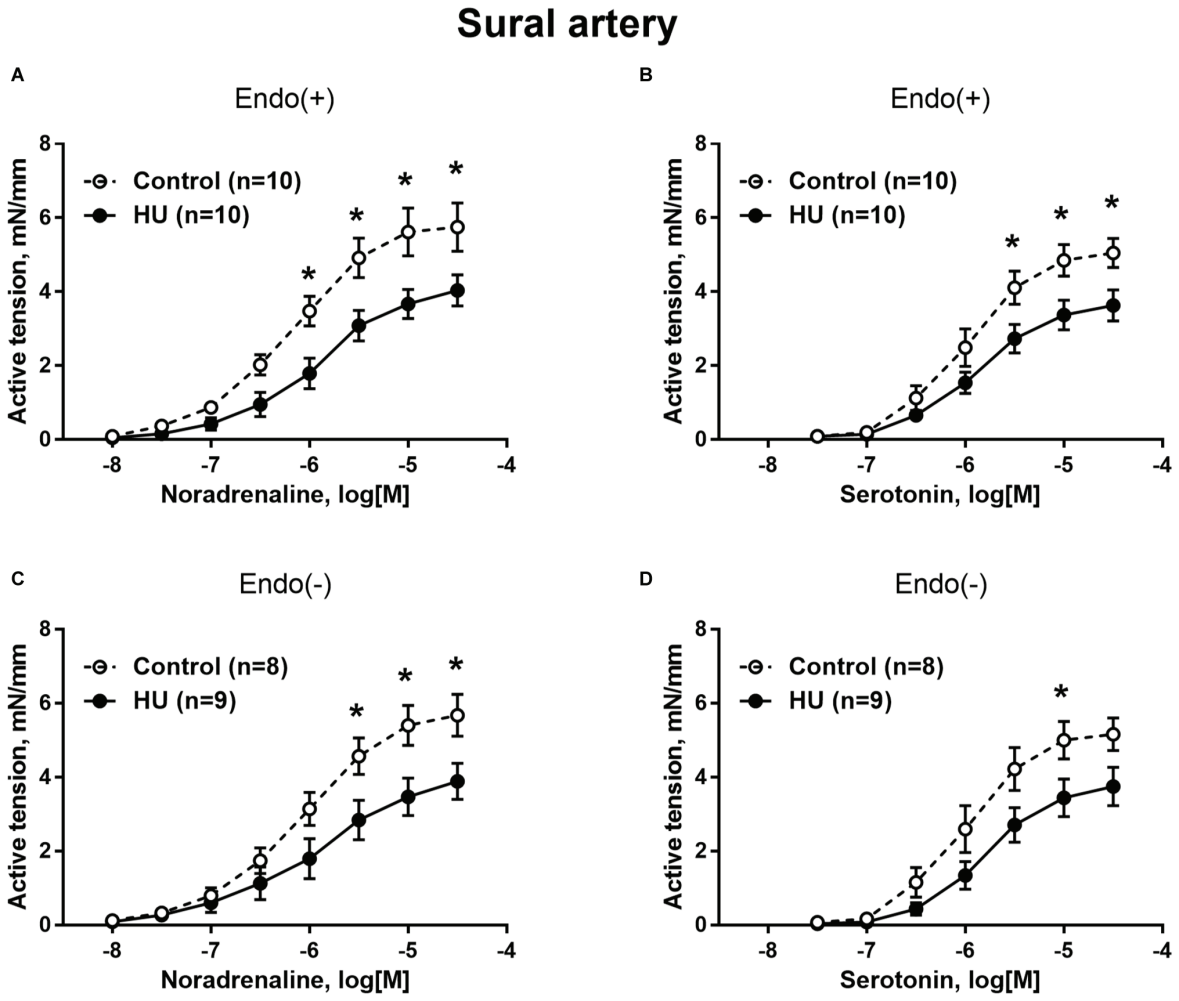
HU decreases contractile responses of skeletal muscle feed arteries from the rat hindlimb. Concentration-response relationships to noradrenaline **(A,C)** and serotonin **(B,D)** of endothelium-intact **(A,B)** and endothelium-denuded **(C,D)** sural arteries in control and HU rats. The number in parentheses indicates the number of animals in the group. ^*^ *p* < 0.05 HU vs. control.

**Figure 4:**
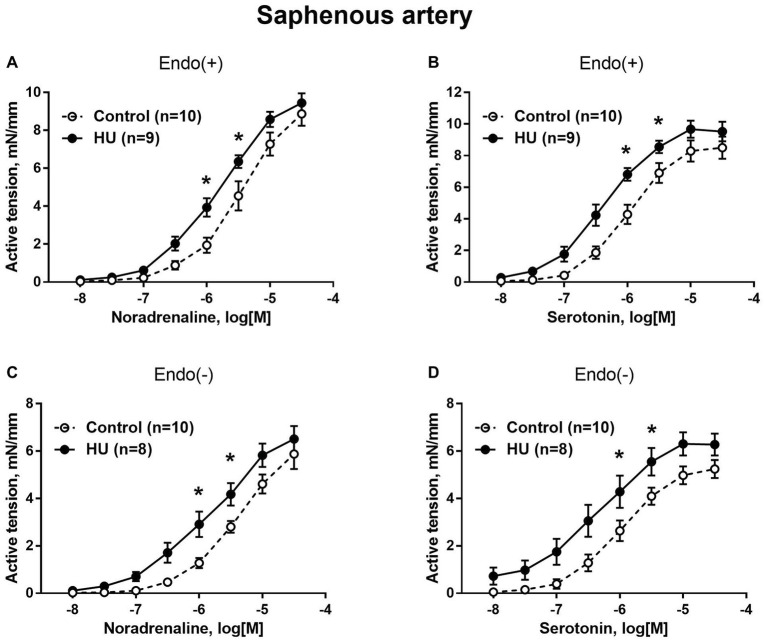
HU increases contractile responses of skin feed arteries from the rat hindlimb. Concentration-response relationships to noradrenaline **(A,C)** and serotonin **(B,D)** of endothelium-intact **(A,B)** and endothelium-denuded **(C,D)** sural arteries in control and HU rats. The number in parentheses indicates the number of animals in the group. ^*^ *p* < 0.05 HU vs. control.

The numbers of animals in the groups are shown in Figures 1–4.

n.d., not determined, because of non-saturated concentration-response relationship.

* *p* < 0.05 HU vs. control.

### Contractile Responses of Forelimb Arteries

Contractile responses of brachial arteries were greater in HU rats compared to that in control rats. This was observed when endothelium-intact segments of brachial arteries were exposed to noradrenaline (Figure 1A) or serotonin (Figure 1B). Endothelium removal did not change the differences in brachial artery contractile responses between HU and control rats (Figures 1C,D). Most prominent effects of HU on brachial artery contractility were observed when the agonists were applied in near-maximal to maximal concentrations. The sensitivity of endothelium-intact and endothelium-denuded brachial artery preparations to vasoconstrictors was not altered by HU (Table 2).

Contractile responses of median artery segments to noradrenaline and serotonin were not different in HU rats compared to control rats regardless of the presence of the endothelium (Figure 2). The sensitivity of median artery segments to both vasoconstrictors was also similar in control and HU rats (Table 2).

### Contractile Responses of Hindlimb Arteries

Sural arteries of HU rats developed less contractile tension to noradrenaline and serotonin. This decrease was observed in both endothelium-intact (Figures 3A,B) and endothelium-denuded (Figures 3C,D) segments of sural artery from HU rats compared to control rats. Again, most prominent effect of HU on contractile responses was observed with the higher concentrations of the agonists, while the sensitivity of sural arteries to contractile stimuli was not altered by HU (Table 2).

In contrast to sural arteries, saphenous arteries from HU rats developed stronger contractile responses compared to the arteries from control rats, regardless of the type of agonist and the presence of the endothelium (Figure 4). HU did not alter saphenous artery maximum contractile responses to noradrenaline and serotonin (Figure 4), but rather elevated the sensitivity of this artery to vasoconstrictors (Table 2).

### Sympathetic Neurotransmission in Saphenous Artery

**Figure 5:**
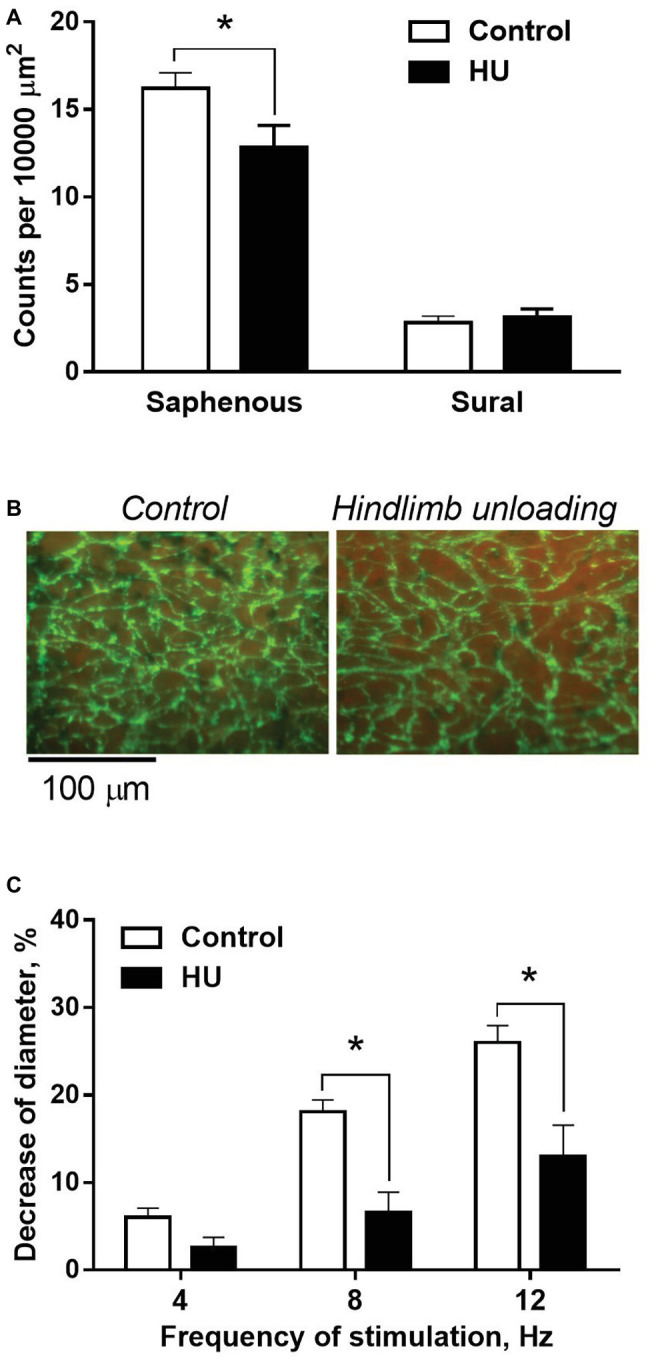
HU worsens sympathetic control in skin but not skeletal muscle feed arteries from rat hindlimb. **(A)** The density of periarterial adrenergic plexus in saphenous and sural arteries from control (*n* = 10; 10) and HU (*n* = 8; 7) rats. **(B)** Representative staining for adrenergic nerves with glyoxylic acid in whole-mount preparations of saphenous arteries. **(C)** Constrictor responses of isobarically perfused saphenous arteries from control (*n* = 5) and HU (*n* = 5) rats to electrical field stimulation of intramural nerves with graded frequencies. ^*^ *p* < 0.05 HU vs. control.

In control rats, adrenergic fiber plexus was dense in the saphenous artery, but sparse in the sural artery (Figure 5A). HU had no effect on innervation density in the sural artery (Figure 5A) but decreased it by about 20% in the saphenous artery (Figures 5A,B).

The effects of HU on sympathetic control of the saphenous artery were studied with the use of perfusion technique. In the absence of nerve stimulation, solution flow rate values did not differ in control and HU rats: 3.9 ± 0.18 and 4.1 ± 0.18 ml/min, respectively. The relative decreases of arterial inner diameter caused by 8 and 12 Hz stimulation were lower in HU rats compared to that in controls (Figure 5C).

## Discussion

The purpose of this study was to compare the effects of 2 weeks of simulated microgravity on skeletal muscle and skin feed arteries from the rat forelimb and hindlimb regions. We hypothesized that HU exerts diverse effects on the sympathetic innervation density, structural properties, and functional vasoconstrictor responsiveness of skeletal muscle and cutaneous feed arteries. The data indicate that HU induces increases in maximal lumen diameter and vasoconstrictor responsiveness in forelimb skeletal muscle arteries, but decreases in these structural and functional properties in hindlimb skeletal muscle arteries. In the cutaneous vasculature of the forelimbs and hindlimbs, alterations in the structure and vasoconstrictor properties either did not occur or were of smaller magnitude than that occurring in the skeletal muscle arteries from the same region of the body. Exposure to HU also diminished sympathetic nerve density and impaired vasoconstriction induced by sympathetic nerve stimulation in hindlimb cutaneous, but not skeletal muscle arteries. However, vasoconstrictor responses of hindlimb cutaneous arteries to direct smooth muscle stimulation by vasoconstrictor agonists exhibited greater sensitivity in the HU rats. Collectively, these results demonstrate that the complexity of vascular adaptations to simulated microgravity is much greater than previously described. Vascular alterations not only differentially occur between regions of the body, but also within those regions, and can be functionally related to physical alterations in sympathetic nerve density and gross structural remodeling of resistance arteries.

Both the forelimbs and hindlimbs of the rat perform functions of support and locomotion, which suggest these two different anatomical regions of the body share similar inherent characteristics that include skeletal muscle and cutaneous circulations. Additionally, systemic humoral influences would not differ in forelimb and hindlimb arteries of the same rat. Alterations in nerve traffic during HU exposure would also likely be equalized between the same-type arteries in the forelimbs and hindlimbs, since functional specialization of efferent sympathetic pathways is organotypic rather than somatotopic (Jänig and McLachlan, 1992; Jänig and Häbler, 2003). Therefore, the design of this study allowed us to separate adaptive arterial responses to the anti-orthostatic body position and redistribution of support and muscle activity from other effects associated with the HU model, including social isolation, restraint, and the development of neuroendocrine stress (Tsvirkun et al., 2012).

### Hindlimb Unloading Induces Contrasting Structural and Functional Adaptations in Forelimb and Hindlimb Skeletal Muscle Arteries

HU exposure resulted in divergent alterations of forelimb and hindlimb skeletal muscle arteries, i.e., lumen diameter and wall contractility (maximal active tension) increased in the forelimb but decreased in the hindlimb. The changes in contractility were independent of the vascular endothelium and not associated with the change in smooth muscle sensitivity to contractile stimulation by noradrenaline or serotonin. These data point to non-specific amplification of smooth muscle contractile responses in forelimb arteries, presumably resulting from smooth muscle hypertrophy and media wall thickening. In contrast, non-specific attenuation of smooth muscle contractile responses takes place in hindlimb arteries, presumably resulting from smooth muscle atrophy and a thinning of the medial wall (Delp et al., 2000).

Previous work has shown that HU in rats is associated with ~10 mmHg gradient between aortic and hindlimb arterial pressures (Colleran et al., 2000). Therefore, arterial transmural pressure in HU rats is elevated in the forelimb region and reduced in the hindlimbs; this pressure differential presumably governs the divergent alterations in media thickness (Wilkerson et al., 1999; Stabley et al., 2012). The mechanisms which mediate the effects of transmural pressure on media remodeling may include the secretion of angiotensin II from vascular smooth muscle cells with further stimulation of MAPK cascades (Eskildsen-Helmond and Mulvany, 2003). Importantly, HU was shown to stimulate the activity of vascular renin-angiotensin system in cranial arteries, but depress it in femoral arteries (Bao et al., 2007; Zhang, 2013). In support of the present data, wall thickness and medial cross-section area were shown to be diminished in resistance arteries associated with the anterior tibial (Sun et al., 2004) and gastrocnemius (Delp et al., 2000) muscles in the hindlimbs of HU rats. In addition, chronic increases in blood flow/shear stress in loaded forelimb muscles and chronic decreases in unloaded hindlimb muscles induce divergent changes in maximal passive diameter of arteries (Delp et al., 2000; Stabley et al., 2013). Similar results have also been found in human models used to simulate microgravity. For instance, long-duration (up to 60 days) head-down bed rest has been reported to reduce intimal-medial thickness in the anterior tibial artery (Platts et al., 2009), as well as lumen diameter of the femoral artery and its branch (Bleeker et al., 2005). Therefore, these results indicate that the gross structural adaptations of skeletal muscle arteries in HU rats and humans undergoing head-down bed rest are mainly induced by the influence of mechanical forces.

In both the forelimbs and hindlimbs, cutaneous arteries were less affected by HU, as compared to skeletal muscle arteries from the same region of the body. These results may be attributed to several factors. First, cutaneous blood flow would not be as greatly affected by changes in postural support and low level physical activity as that of the skeletal muscle, particularly given the rapid and profound changes in skeletal muscle vascular resistance associated with increases and decreases in muscle activity (Delp, 1999). Second, the profile of blood pressure changes within the cutaneous vasculature may differ from that within the skeletal muscle vascular tree. A prominent constriction of proximal cutaneous arteries during sympathetic activation (Tarasova et al., 2003) would decrease transmural pressure in more distal arteries (Abboud and Eckstein, 1966) and protect them from redistribution of transmural pressure during HU.

### Hindlimb Unloading Had Diverse Effects in Two Functionally Different Vascular Beds of the Rat Hindlimb

In the hindlimb, 2-week exposure to HU impaired sympathetic nerve-mediated vasoconstriction while enhancing the sensitivity of smooth muscle to the adrenergic agonist noradrenaline in cutaneous arteries. The higher constrictor sensitivity of cutaneous arteries was also observed after endothelium removal and with the application of the non-adrenergic agonist serotonin, pointing to its independence of the type of activated receptors in smooth muscle cells. These data suggest the development of non-specific hyper-sensitivity of the saphenous artery to constrictor stimulation, which may result from the deficiency in its sympathetic control. This suggestion is based on several observations from the present and earlier published studies: (1) the higher sensitivity to constrictors was observed in densely innervated saphenous artery but not in the sparsely innervated sural artery; (2) HU reduced the density of adrenergic plexus in saphenous arteries; and (3) denervated vessels are well-known to become hyper-sensitive to various constrictor agonists and high-K^+^ depolarization (Bevan, 1984; Bentzer et al., 1997; Tarasova et al., 2006). Post-denervation hyper-sensitivity occurs without changes in total density or affinity of post-junctional α_1_-adrenoceptors (Dalessandri et al., 1991; Taki et al., 2004), indicating its mechanisms of effect are downstream to the receptors and may include depolarization of smooth muscle cells (Fleming, 1999), facilitation of L-type Ca^2+^-channel-dependent signaling (Heumann et al., 2016), Ca^2+^-sensitization of smooth muscle contractile machinery (Puzdrova et al., 2014), and altered cell-to-cell coupling (Slovut et al., 2004). Taken together, these observations allow the conclusion that the shortage of sympathetic influences may govern functional remodeling of arteries subjected to gravitational unloading.

In our opinion, the effect of HU on saphenous artery sympathetic innervation is due in part to a drop in transmural pressure in hindlimb arteries during long-term anti-orthostasis (Colleran et al., 2000). It is well-known that the functional state of postganglionic nerve fibers and the density of sympathetic innervation is controlled by target tissue-derived trophic factors, including nerve growth factor (NGF) (Creedon and Tuttle, 1991; Thrasivoulou and Cowen, 1995). For example, it has been shown that NGF secretion by vascular smooth muscle cells in cell culture increases with stretch (Clemow et al., 2000). Presumably, the production of NGF under *in vivo* conditions is also dependent on the stretch induced by the transmural pressure. A chronic decrease in transmural pressure in the saphenous artery of HU rats could suppress NGF production and, therefore, result in the decrease of adrenergic nerve plexus density. We have shown previously that chronic hindquarter hypotension (induced by partial occlusion of the abdominal aorta distal to the renal arteries) has negative effects on sympathetic innervation of the rat saphenous artery (Tarasova et al., 2006). Interestingly, an opposite change of innervation density was demonstrated in the rat saphenous artery after the 2-week experimental orthostatic body position (Monos et al., 2001). Results from the present study thus indicate that chronic decreases in transmural pressure worsens sympathetic vasomotor control at the prejunctional level, but at the same time, increases vessel reactivity to noradrenaline, a principal transmitter of the sympathetic nerves. Further work should be conducted to determine whether noradrenaline release during activation of the sympathetic nerves in skin arteries differs between control and HU rats to better characterize how sympathetic neural control of peripheral resistance is altered.

In contrast to cutaneous arteries, we did not find any effect of transmural pressure changes on the adrenergic nerve plexus in the sural artery, probably due to the already low density of its innervation in control rats. However, we suggest that such effects of pressure to decrease the innervation density at distal part of skeletal muscle arterial tree may occur, since the more distal resistance arteries have a higher density of innervation. Indeed, a decrease in the innervation density was observed in hindlimb muscle arterioles in rats after a 4-week HU (Zhang, 2001). This suggestion is supported by our findings of a reduced integral response of perfused rat hindlimb vascular bed to the stimulation of sympathetic nerves (Rodionov et al., 1999).

Our findings indicate that the precision in which the sympathetic nervous system controls peripheral resistance and cutaneous perfusion is compromised in HU rats. Importantly, a decreased vascular sensitivity to sympathetic influences has been observed in humans following space flight (Fu et al., 2002). A smaller increase in lower limb arterial resistance was observed in cosmonauts during infight and postflight lower body negative pressure (LBNP) test (Herault et al., 2000). In contrast, forelimb subcutaneous vascular response to LBNP was more pronounced after 10-day-long space flight than during preflight (Gabrielsen et al., 1995). Although the present study is limited to an animal model used to simulate spaceflight, it provides a mechanistic explanation of the link between the state of sympathetic vascular control with the prevailing changes in transmural pressure thought to occur in astronauts and cosmonauts.

## Conclusion

A novel result of this study is that simulated microgravity differentially affects the skin and skeletal muscle arteries in the hindlimb. Functional alterations in the vasoconstrictor responses of sparsely innervated skeletal muscle arteries appear to primarily result from the direct effects of physical forces acting on smooth muscle or endothelial cells to induce a gross structural remodeling of these arteries. However, functional changes in the densely innervated cutaneous arteries appear to be mainly governed by the effect of transmural pressure on periarterial sympathetic innervation. If similar adaptations occur in the peripheral vasculature of human cosmonauts and astronauts, such changes could underlie the weaker arterial vasoconstriction during activation of the sympathetic nerves, as well as serve as a mechanism of reduced vascular resistance and orthostatic intolerance following a return to Earth.

## Data Availability Statement

The datasets generated for this study are available on request to the corresponding author.

## Ethics Statement

The animal study was reviewed and approved by Biomedical Ethics Committee of the Institute of Biomedical Problems, Russian Academy of Sciences.

## Author Contributions

OT, AB, VG, MD, and OV conceived and designed the study. OT, VK, AB, and VG were involved in laboratory work, data collection, and analysis. OT, AB, MD, and OV drafted the manuscript. All the authors contributed in the final writing.

## Conflict of Interest

The authors declare that the research was conducted in the absence of any commercial or financial relationships that could be construed as a potential conflict of interest.
